# The Relationship between Dehydroepiandrosterone (DHEA), Working Memory and Distraction – A Behavioral and Electrophysiological Approach

**DOI:** 10.1371/journal.pone.0104869

**Published:** 2014-08-08

**Authors:** Sónia do Vale, Lenka Selinger, João Martin Martins, Ana Coelho Gomes, Manuel Bicho, Isabel do Carmo, Carles Escera

**Affiliations:** 1 Institute for Brain, Cognition and Behavior (IR3C), University of Barcelona, Barcelona, Catalonia, Spain; 2 Cognitive Neuroscience Research Group, Psychiatry and Clinical Psychobiology Department, University of Barcelona, Barcelona, Catalonia, Spain; 3 Endocrinology University Clinic, Lisbon Medical School, University of Lisbon, Lisbon, Portugal; 4 Endocrinology, Diabetes and Metabolism Department, Santa Maria University Hospital, Lisbon, Portugal; 5 Metabolism and Endocrinology Center, Genetics Laboratory, Lisbon Medical School, University of Lisbon, Lisbon, Portugal; University Medical Center Groningen UMCG, Netherlands

## Abstract

Dehydroepiandrosterone (DHEA) and dehydroepiandrosterone-sulphate (DHEAS) have been reported to have memory enhancement effects in humans. A neuro-stimulatory action and an anti-cortisol mechanism of action may contribute to that relation. In order to study DHEA, DHEAS and cortisol relations to working memory and distraction, we recorded the electroencephalogram of 23 young women performing a discrimination (no working memory load) or 1-back (working memory load) task in an audio-visual oddball paradigm. We measured salivary DHEA, DHEAS and cortisol both before each task and at 30 and 60 min. Under working memory load, a higher baseline cortisol/DHEA ratio was related to higher distraction as indexed by an enhanced novelty P3. This suggests that cortisol may lead to increased distraction whereas DHEA may hinder distraction by leading to less processing of the distractor. An increased DHEA production with consecutive cognitive tasks was found and higher DHEA responses attributed to working memory load were related to enhanced working memory processing as indexed by an enhanced visual P300. Overall, the results suggest that in women DHEA may oppose cortisol effects reducing distraction and that a higher DHEA response may enhance working memory at the electrophysiological level.

## Introduction

Dehydroepiandrosterone-sulphate (DHEAS) is the most abundant steroid in the peripheral circulation and it is much more abundant in humans than in any other species [1], [2], [3], [4], [5], [6], [7]. Circulating levels dramatically decrease with aging. Moreover, lower levels are related to higher morbidity and mortality ratios even when corrected for age [2], [8].

DHEA is mostly synthesized in the adrenals whereas the gonads represent a minor source of this hormone. However, DHEA is also synthesized in the central nervous system [3], where its concentrations are higher than in the peripheral circulation [3], [9], [10]. In both peripheral and central compartments a sulpho-transferase reversibly converts DHEA to DHEAS, restricting its distribution and prolonging its half-life [2], [3].

Several central effects concerning cognitive performance and stress response have been described for DHEA and DHEAS. Higher levels were related to increased memory and attention scores [9], [11] and improved performance in stressful conditions [12], [13], [14], whereas low levels were found in Alzheimer’s disease [15]. However, DHEA administration in older subjects showed inconclusive results [5], [16], [17], [18], [19].

On the other hand, stressful stimuli also modulate DHEA and DHEAS levels: acute stress is related to an increase in DHEA and DHEAS levels [20], [21], [22], [23] whereas chronic stress decreases baseline DHEA and DHEAS levels [24], [25], [26], [27], [28], as well as the acute DHEAS response to a superimposed psychological stress [29]. Yet, a direct influence of cognitive processing on DHEA or DHEAS levels has not been studied.

At the molecular level, DHEA and DHEAS have a general neuro-stimulatory effect: presynaptic actions include glutamate, acetylcholine and norepinephrine release and postsynaptic actions include sigma 1 receptor agonism with subsequent *N*-methyl-D-aspartate (NMDA) receptor activation, gabaminergic antagonism and inhibition of voltage-gated calcium currents [3], [9], [10]. Nevertheless, DHEA and DHEAS molecular effects are not exactly the same and several studies suggest that the balance between them may influence brain functioning [9], [30]. As an example, DHEAS has more potent antagonistic effects at the γ-aminobutyric acid type A receptor (GABA_A_ receptor) than DHEA [31], [32]. Hence, the simultaneous evaluation of DHEA and DHEAS may uncover more information than the individual examination of either form of that steroid.

Concerning glucocorticoids, whereas mild or short-lasting increases in cortisol due to stress may protect the body, promote adaptation and have beneficial effects on attention and memory, higher cortisol levels or long term increases are related to poorer executive functioning, poorer learning and memory and less cognitive flexibility [33], [34], [35], [36], [37], [38], [39]. In particular, working memory (WM) depends on prefrontal cortex activity, which is modulated by glucocorticoids: prefrontal cortex-dependent working memory is enhanced by acute stress and inhibited by chronic stress [35], [40]. The relation between glucocorticoids and cognitive functioning is bidirectional: glucocorticoids impact cognitive function and cognitive processing has been shown to influence glucocorticoid secretion [41].

Several levels of evidence suggest that DHEA and DHEAS may counterbalance cortisol effects: a higher DHEA/cortisol ratio has been related to better performance under stress [14] and DHEAS antagonized the memory deteriorating neurotoxic effects of cortisol in the hippocampus [10], [42]. More generally, DHEA and DHEAS decrease with aging, whereas cortisol does not. Consequently, the cortisol/DHEA ratio increases and may be involved both in the cognitive impairments and in the particular vulnerability to stress damage that seems to characterize the elderly [5], [9], [42].

The aim of the present study was to test whether DHEA and DHEAS levels are modulated by WM load and whether these endocrine levels are related to distraction and WM at the electrophysiological level, as evidence for their neurophysiologic effects using Event-Related Brain Potentials (ERPs) is scarce [43], [44], [45]. The specific *a priori* hypotheses were: 1) higher endogenous DHEA and DHEAS levels may prevent involuntary distraction and enhance cognitive performance; 2) DHEA and DHEAS putatively beneficial effects may be translated at the neurophysiological level; 3) DHEA and DHEAS effects may be largely antagonistic from those of high baseline cortisol; and 4) WM load may be a stimulus for DHEA and DHEAS production.

To test these hypotheses we measured the relation between DHEA, DHEAS and cortisol on one hand and the cognitive performance and brain responses, using a well-established auditory-visual distraction paradigm [46], [47], [48] on the other. The protocol includes task irrelevant sounds, some of which are aimed to cause distraction and a visual task including working memory manipulation.

## Subjects and Methods

### Ethics Statement

The experimental protocol was approved by the ethical committees of the University of Barcelona and Lisbon Medical School and it was conducted according to the principles of the Declaration of Helsinki. All the subjects gave their written informed consent before entering the study.

### Subjects

28 healthy female volunteers (undergraduated Psychology students) performed the study protocol. In order to ensure a higher homogeneity in androgen levels, only women were included. The subjects were young (18 to 25 years old, mean 20±0.5 years old) and presented a normal body mass index (21.8±0.5 kg/m^2^). All the participants had a normal or corrected-to-normal vision and none reported auditory deficits. None of the participants reported a past history of neurologic, psychiatric, endocrine or oral diseases. All the subjects were right-handed. Four subjects were under hormonal contraception. No other medications were allowed. Regular or binge alcohol consumption were exclusion criteria and subjects were asked not to consume alcohol in the twelve hours before the experimental protocol. Regular tobacco consumption as well as illicit drug consumption were further exclusion criteria. Prior to the experimental session, subjects completed the State-Trait Anxiety Inventory [49] and all showed a normal range of state and trait anxiety levels. Five subjects were discarded from the analysis due to technical problems with electroencephalogram (EEG) recordings or endocrine measurements.

### Task and Procedure

The experimental sessions were held in the afternoon, beginning at 2–3 pm. An adapted version of a well-established auditory-visual distraction task [46], [47], [50] was presented, based on the protocol used by SanMiguel et al. (2008) [51]. In this protocol, two visual tasks were performed: one task without working memory load (WM0) and another one with working memory load (WM1). In the present experiment, the two tasks were performed two hours apart from each other (from onset to onset) and the order of the tasks was counterbalanced across participants. Each task lasted about 15 minutes, and consisted of two blocks of 250 trials each (plus five initial trials that were excluded from the analyses). A short pause was allowed between blocks.

**Figure 1:**
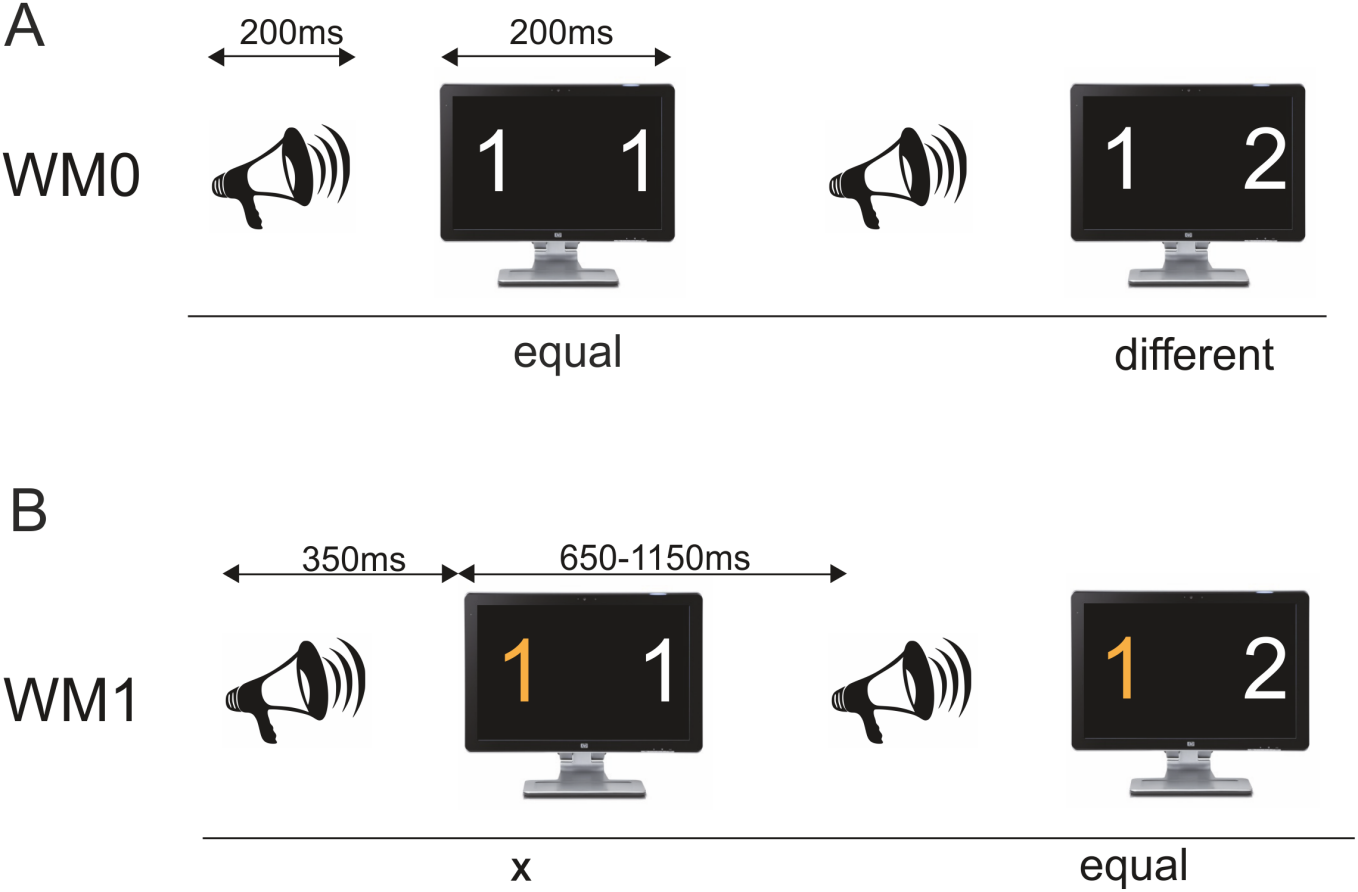
Example of trials stimulation sequence (above the line) and correct answers to the tasks (below the line) for the two tasks. A) Discrimination task (WM0), in which subjects had to decide whether the two digits on the screen were equal or different. B) Working memory task (WM1), in which the subjects had to compare the left digit on the screen with the left digit of the previous trial. WM0 – discrimination task; WM1 – working memory task.

Participants sat in a comfortable chair in a dimly lit and electrically and acoustically shielded room. In the discrimination task (WM0) subjects had to decide whether the two digits appearing on the screen were the same (11 and 22) or different (12 and 21), see figure 1A. In the WM1 task (1-back task) subjects had to decide whether the left or right digit (counterbalanced across subjects) on the screen was the same as the left or right digit of the previous trial (figure 1B). Thus, they had to keep one digit in working memory until the next trial, answering to every trial, except for the first one. Responses were given through a mouse button (one mouse button for “same” and the other button for “different”), also counterbalanced across subjects. The subjects were specifically instructed to respond as quickly and accurately as possible while ignoring the sounds. In order to reduce artifacts originating from eye-blinks and movements during EEG recording, subjects were asked to minimize blinking and to focus on a central fixation cross between the two digits. Before each task, subjects performed practice blocks (composed by 10 trials) without any auditory stimuli until they reached a hit rate of at least 80% in each task.

Each trial consisted of an auditory stimulus, irrelevant for the task, followed by a visual imperative stimulus after 350 ms (onset to onset), see figure 1. The total trial length varied from 1000 to 1500 ms (1250 ms on average; jitter +/–250 ms). The auditory sequence consisted of repetitive standard tones (200 ms, including fade-in and fade-out of 10 ms each; 600 Hz; 85 dB; 80% probability), occasionally replaced by environmental novel sounds selected from a sample of 100 different exemplars (edited to have a duration of 200 ms, including fade-in and fade-out of 10 ms each; digitally recorded, low-pass filtered at 10,000 Hz; 85 dB; 20% probability), such as those produced by a drill, hammer, rain, door, telephone ringing, and so forth (50). All sounds were randomly delivered binaurally through headphones (Sennheiser HD 202), and the only restrictions were that the first four stimuli of each block were standard tones, that two novel sounds never appeared consecutively and that each novel sound occurred only once in each task. The visual stimuli consisted of pairs of combinations of the digits 1 and 2 (11, 12, 21 or 22), presented on a computer screen for 200 ms. The appearance probability was the same for every digit combination. The picture size was 357×441 pixels, with a vertical angle of 8° and a horizontal angle of 18°, accounting for two pictures presented simultaneously with the fixation cross in between. The distance from the subjects’ eyes to the screen was 100 cm. Overall, there were 400 standard trials and 100 novel trials in each of the two WM conditions.

We recorded response time and hit rates for each trial with Presentation® (Neurobehavioral Systems, Inc). A correct response within the response window (until the sound onset of the subsequent trial) was counted as a hit. We computed distraction as the difference in hit rate or response time between auditory stimulus types (hit rate: WM0standard trials − WM0novel trials and WM1standard trials − WM1novel trials; response time: WM0novel trials − WM0standard trials and WM1novel trials − WM1standard trials) and working memory load costs as the difference in hit rate or response time between the WM load and the discrimination task (hit rate: WM0standard − WM1standard and WM0novel − WM1novel; response time: WM1standard − WM0standard and WM1novel − WM0novel).

### EEG Recording and Analysis

Electrophysiological activity was continuously recorded during task performance, from 64 scalp Ag/AgCl electrodes following the extended 10/10 convention. Elastic caps with sintered electrodes and shielded wires were used. The horizontal and vertical electrooculograms (HEOG and VEOG) were recorded with electrodes placed at the outer canthus and below the right eye, respectively. An electrode placed on the tip of the nose was used as the common reference and the ground was located at the AFz position. The EEG and electrooculogram (EOG) were amplified and digitized at a sampling rate of 512 Hz (Eemagine, ANT Software b.v., Enschede, the Netherlands). Impedances were kept at 5 kΩ or below during the whole recording session. Recording was performed with an ANT amplifier of 64 channels (gain 20x; A/D resolution 22 bits, 71.526 nV per bit; filtering 0–138.24 Hz; CMRR>90 dB).

A digital finite impulse response (FIR) bandpass-filter from 0.01 to 30 Hz was applied using a Hamming window. ERPs were averaged offline for each auditory stimulus type and working memory condition, for an epoch of 1000 ms, including a 200 ms pre-auditory-stimulus baseline. The first five epochs of each block and the epochs following a novel trial were excluded from averaging. Only epochs that corresponded to trials with correct responses were averaged.

EOG correction was performed by manually selecting a large number of typical artifacts and accordingly applying a regression algorithm to compute propagation factors (Eeprobe 3.1, ANT Software BV, Enschede, the Netherlands). After EOG correction, epochs that contained EEG activity exceeding ±100 µV peak-to-peak amplitudes were rejected from further analyses. Since we included only trials with correct answers and hit rate was smaller for WM1, the final number of trials was smaller for WM1. The total number of trials included in the averages for each condition and auditory stimulus type was: 246 trials with standard sounds and 81 with novel sounds in WM0 and 213 trials with standard sounds and 64 trials with novel sounds in WM1.

In this paradigm, subjects are specifically instructed to ignore the sounds. Hence, any related effects are necessarily involuntary or lead by exogenous attention. ERPs recorded during this auditory-visual distraction paradigm typically present first an auditory N1/mismatch negativity (N1/MMN) enhancement, reflecting a detection mechanism that leads to attention capture [46], followed by a novelty-P3 (nov-P3) that reflects the effective attention orientation [46], [47], [52]. Finally, the re-orienting negativity (RON) reflects the attention re-orientation back to the task [53]. The target is visual and visual ERPs yield sensory (visual P1 and N1) and cognitive components related to target processing (N2b and P300). The N2b is a negative deflection originated by the relevant stimulus and the P300 reflects the processing of the task-relevant visual stimulus [51], [54], [55]. Typically the task with working memory load is more difficult for the subjects, leading to a reduced P300 [51], [54], [55].

To analyze distraction effects at the electrophysiological level, difference waves (dw) were calculated by subtracting the ERPs elicited in standard trials from those elicited in novel trials. These difference waves revealed an early-onset, long-lasting positive deflection that we assimilated to the novelty-P3. Novelty-P3 was measured as the mean amplitude in a time window ranging from 250 to 380 ms at F3, Fz, F4, C3, Cz, C4, P3, Pz and P4 electrodes.

To analyze WM effects we compared ERP measures only in standard trials. Specific auditory and visual components were elicited during task-performance: the auditory N1 and P2 and visual P1 and N1. Yet, since cognitive processing was of interest for the present study, we only analyzed N2b (560–645 ms, 210–295 ms from visual stimulus presentation) and P300 (650–910 ms, 300–560 ms from visual stimulus presentation): F3, Fz, F4, C3, Cz and C4 for N2b and P3, Pz and P4 for P300. Moreover, since the P300 latency was different across WM conditions [F_(1,21)_ = 5.683, p = 0.027; 753±62 ms for WM1; 787±36 ms for WM0], we analyzed the amplitude of this component at different time windows for each condition (WM1: 650–875 ms; WM0-670–910 ms).

### Endocrine Measurements

We collected saliva samples by means of passive drool, using a short straw. Unstimulated whole saliva was used. We collected samples for DHEA, DHEAS and cortisol measurement before each task [before task (BT)], at 30 min [after task (AT)] and 60 min [washout (WO)]. The samples collected before the first task (i.e. before both tasks) were considered as the baseline. We chose the time points to collect the saliva samples in accordance to known cortisol raise and recovery times-raise 10 min after appropriate stimulus, peak at 20–30 min and recover at 45–60 min. Furthermore, synchronous 24 h profiles were described for DHEA and cortisol [56].

Unbound DHEA and cortisol in the peripheral circulation penetrate into the saliva via intracellular mechanisms and salivary concentrations reflect serum concentrations [57], [58]. DHEAS is not lipid soluble and cannot penetrate into the saliva by passive diffusion through cell membranes. Instead, it squeezes through the tight junctions between salivary glands. DHEAS concentrations in saliva are therefore dependent on serum concentration and salivary flow rate [57].

Samples were refrigerated at 2–8°C within 30 minutes after collection and they were stored at −20°C within 4 h and until assayed. Each sample was measured in duplicate by using enzyme-linked immunoassays: salivary DHEA and DHEAS enzyme immunoassay kits (Salimetrics Europe®, Ltd, Newmarket Suffolk, UK) and high sensitivity salivary cortisol enzyme immunoassay kits (Salimetrics®, LLC, State College, PA, USA). DHEA was measured in pg/mL and cortisol was measured in µg/dL. Due to the influence of saliva flow rates on DHEAS levels, the concentration of DHEAS (pg/mL) was multiplied by the flow rate (mL/min) and the corrected results were obtained as DHEAS measured per unit of time (pg/min). Intra- and interassay variation coefficients were less than 10% and 15%, in every case, respectively. Cortisol and DHEA were expressed as pmol/L by using the conversion factors 27590 and 0.3467, respectively, and DHEAS was expressed as pmol/h by using the conversion factor 0.16284 (system of international units = conventional units×conversion factor).

### Statistical Analysis

The Statistical Package for the Social Sciences Program (IBM SPSS Statistics, version 21) was used for data analyses. Results are presented as the mean ± standard error of the mean (SEM). The normal distribution of continuous variables was verified by the Kolmogorov-Smirnov Goodness of Fit Test and non-normal distributed variables were log (ln) transformed prior to the analysis. For the sake of simplicity, the results of non-transformed variables are presented whenever we did not find any differences.

To explore the effects of WM load and auditory distraction on performance we performed repeated measures analyses of variance (ANOVAs) on hit rate and response time, including the within-subjects factors task (WM0 and WM1) and sound (standard and novel). Regarding electrophysiological responses, we examined the auditory ERPs to explore distraction effects and the visual ERPs to explore WM effects. To investigate the effects of auditory distraction on ERPs, we carried out repeated measures ANOVAs on the mean amplitude of the auditory P3 in the time window and electrodes considered above, with the within-subjects factors task (WM0 and WM1) and sound (standard and novel). To investigate WM effects on distraction ERPs, we carried out an ANOVA on the mean amplitude of the novelty-P3 in the time window and electrodes considered above with task (WM0 and WM1) as within-subjects factor. To investigate WM effects on electrophysiological responses, we included only standard trials and conducted ANOVAs on N2b and visP300 mean amplitude in the time windows and electrodes considered above, with task (WM0 and WM1) as within-subjects factor and task order (simply referred as order in the results) as between-subjects factor (WM0-WM1 or WM1-WM0; this factor is included because we used a blocked protocol, one task consisting exclusively in WM0 trials and the other consisting exclusively in WM1 trials, counterbalanced across subjects).

To investigate the effects of WM manipulation on endocrine levels, we performed repeated measures ANOVAs on DHEA, DHEAS and cortisol levels, including the within-subjects factors task (WM0 and WM1) and time (before task, after task and washout) and the between-subjects factor task order (WM0−WM1 or WM1−WM0). The endocrine relation to distraction and WM load effects at the behavioral and electrophysiological levels was investigated by repeated measures ANOVAs of behavior and ERP parameters, including baseline DHEA, DHEAS, cortisol or cortisol/DHEA ratio as covariates. Because the DHEA response (determined by the ratio after task/before task) differed between the two WM conditions, DHEA response in WM1 – DHEA response in WM0 (Δ DHEA response) was used as a measure of DHEA response attributed to WM load. This new variable was also tested as a covariate. Whenever the endocrine parameters covariated with the performance or ERP parameters, for each type of auditory stimuli or WM condition, we used Spearman’s Rank Order correlation coefficients to select the relevant endocrine factors and understand the relations direction. DHEAS relations to performance and ERP parameters were not significant and therefore those results are not mentioned in the results section.

ANOVA results were Greenhouse–Geisser corrected whenever the assumption of sphericity was violated. *Post hoc* tests were carried out wherever there were significant interactions between main factors. The Bonferroni correction was used for multiple comparisons. The limit of significance chosen was α = 0.05.

## Results

### Performance

**Figure 2:**
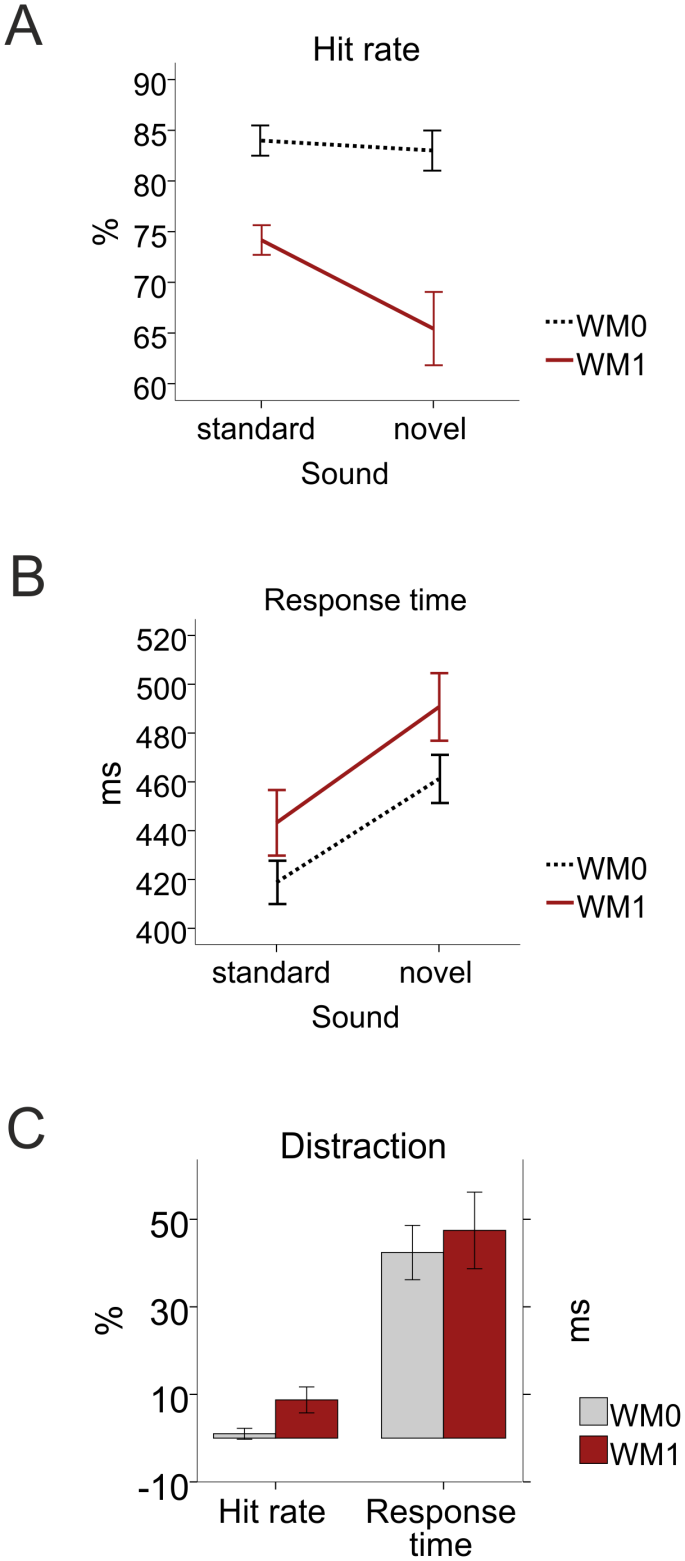
Performance results. A) Mean hit rate for each task and auditory stimulus type. B) Mean response time for each task and auditory stimulus type. C) Distraction costs for each task. Distraction = hit rate in standard minus novel trials or response time in novel minus standard trials. WM0 – discrimination task; WM1 – working memory task. Bars represent +/– standard error of the mean (SEM).

Behavioral results for each task and auditory stimulus are presented in Figure 2. Overall hit rate was 83±2% in WM0 and 70±2% in WM1 (figure 2A) and this difference in hit rate was significant, as reflected by a main effect of task [F_(1,22)_ = 27.279, p<0.001]. Additionally, there was a main effect of task on response times [F_(1,22)_ = 8.760, p = 0.007] with longer response times in WM1 [467±13 ms] than in WM0 [440±9 ms], see figure 2B. Thus, the WM load resulted in lower hit rates and longer response times.

Regarding the effects of auditory distraction on performance, the results showed a main effect of auditory stimulus both on hit rate [F_(1,22)_ = 9.577, p = 0.005, with lower hit rates for novel sounds (74±2%) than for standard ones (79±1%), see figure 2A], and response times [F_(1,22)_ = 43.451, p<0.001, with longer response times for novel sounds (476±11 ms) than for standard ones (431±10 ms), see figure 2B]. This means that novel sounds resulted in auditory distraction reflected by lower hit rates and longer response times.

The interaction between task and auditory stimulus also had notable significant effects on hit rates [F_(1,22)_ = 5.557, p = 0.028]. *Post hoc* analyses on each WM condition yielded significant effects of auditory distraction only in the working memory load task [F_(1,22)_ = 8.697, p = 0.007], see figure 2C. In that condition, hit rates were lower for novel sounds (65±4%) than for standards (74±1%).

### Event-Related Potentials

#### Distraction effects

**Figure 3:**
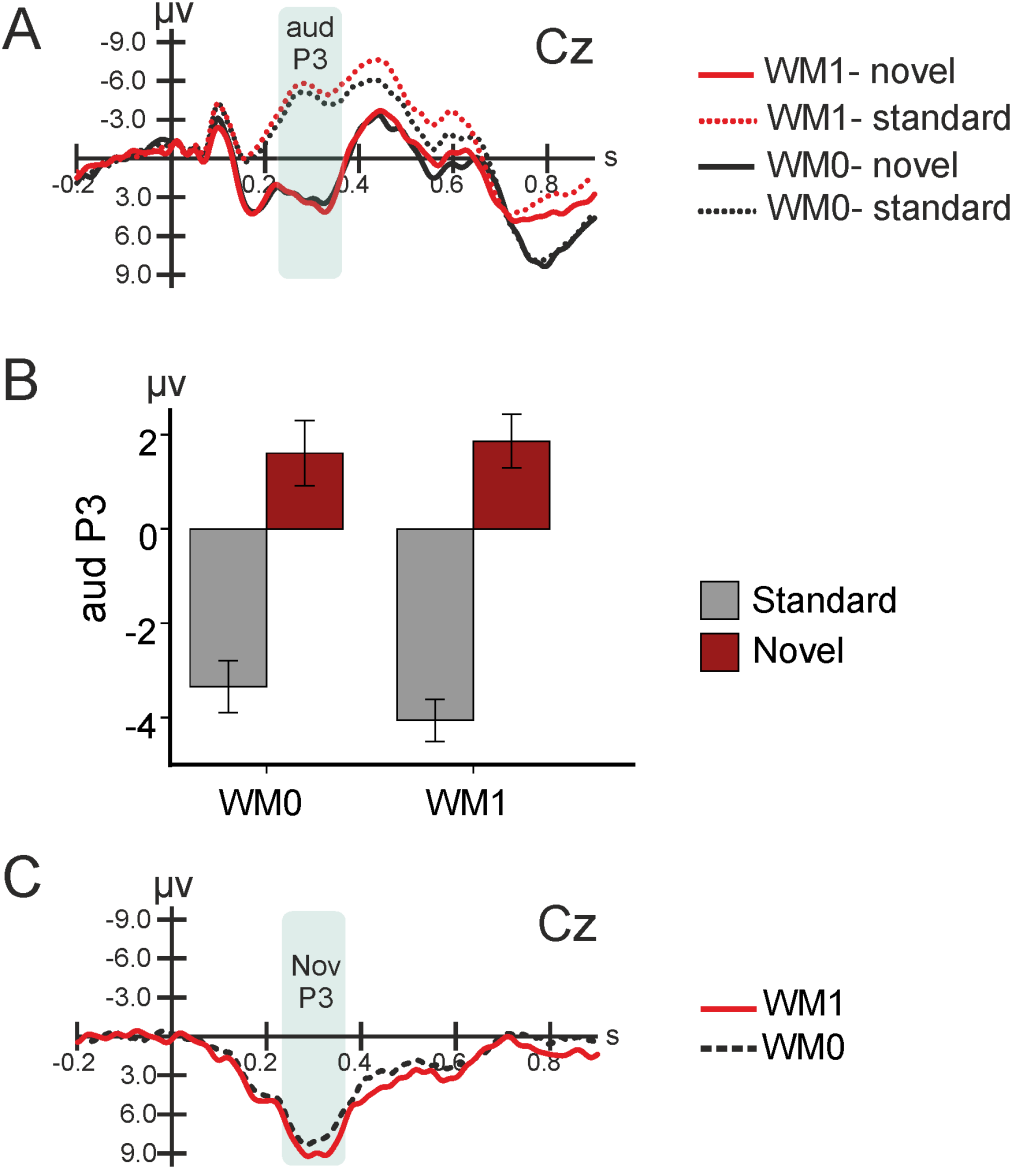
Event Related Brain Potentials (ERPs). A) Grand average waveforms at Cz for both tasks (WM0 and WM1) and type of sound (standard and novel). B) Auditory P3 (aud P3) amplitude for each task (WM0 and WM1) and type of sound (standard and novel). C) Grand-average of novel minus standard difference waves at Cz. WM0 – discrimination task; WM1 – working memory task; NovP3 – novelty P3; s - seconds. Bars represent +/– standard error of the mean (SEM).

As can be seen in figure 3A, novel sounds elicited larger auditory P3 mean amplitudes when compared to standard sounds, both in WM0 [F_(1,22)_ = 63.696, p<0.001; −3.3±0.5 µV in standard and +1.6±0.7 µV in novel trials] and in WM1 [F_(1,22)_ = 98.333, p<0.001; −4.1±0.4 µV in standard and +1.9±0.6 µV in novel trials], see figure 3B. This supports that a significant novelty P3 was elicited by novel sounds (figure 3C). However, WM load did not influence the novelty-P3, as its amplitude was similar in both tasks [F_(1,22)_ = 3.381, p = 0.079, 5.0±0.6 µV in WM0, and 5.9±0.6 µV in WM1]. Neither clear N1-enhancement/MMN nor RON was elicited. In sum, significant novelty-P3 responses were elicited by novel sounds in both conditions, but without any significant difference between conditions.

#### Working Memory Effects

**Figure 4:**
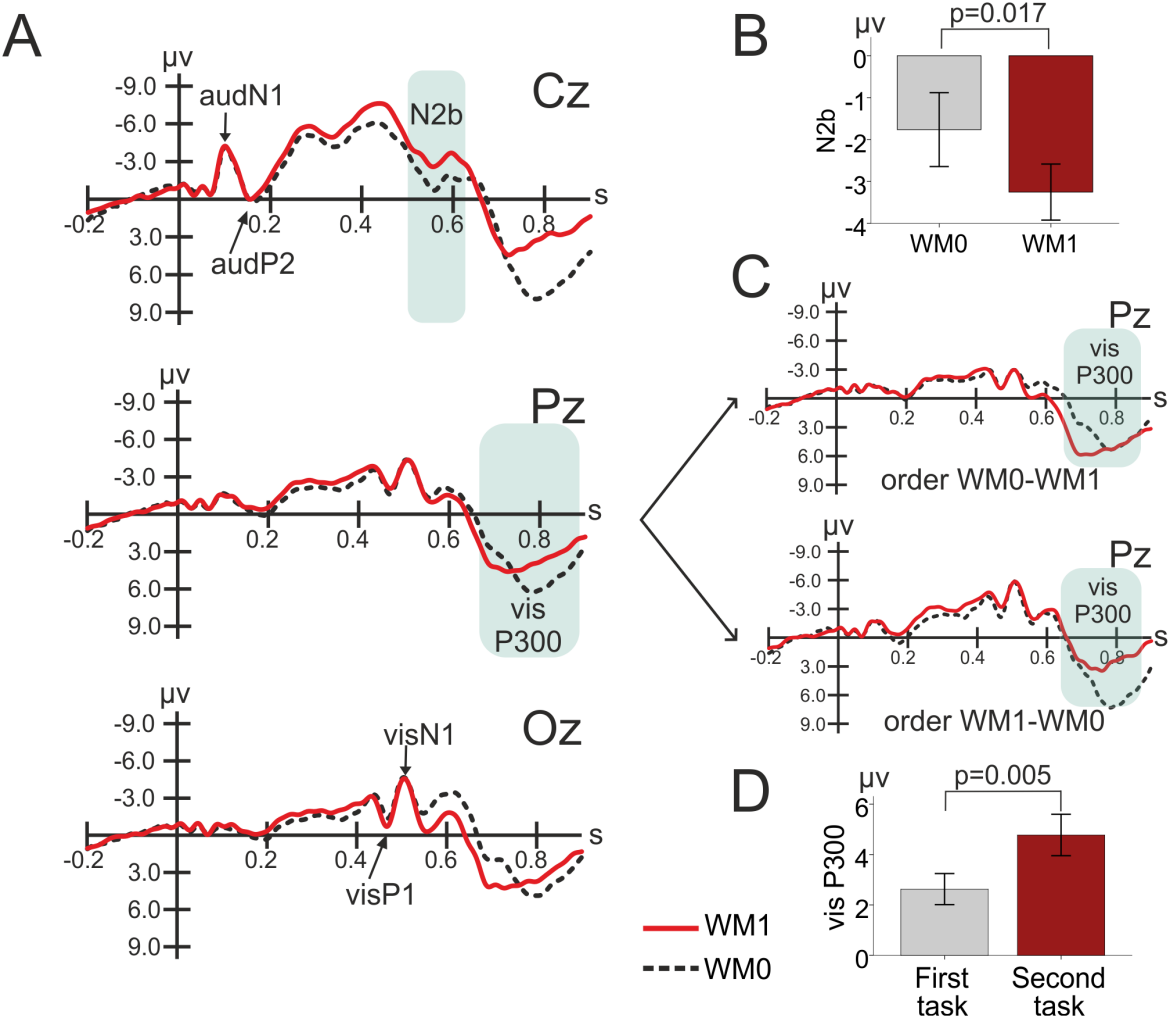
Standard Event-Related Potentials (ERPs) waveforms in the discrimination and working memory tasks. A) Standard ERPs in both tasks (both task orders). B) Mean N2b amplitude for each task. C) Standard ERPs in both tasks at Pz, according to the tasks order. D) Mean visual P300 amplitude according to the temporal sequence of tasks (first and second). WM0 – discrimination task; WM1 – working memory task; s – seconds; order WM0-WM1 – the discrimination task performed firstly and the working memory task performed secondly; order WM1-WM0 – the working memory task performed firstly and the discrimination task performed secondly. Bars represent +/− standard error of the mean (SEM).

**Figure 5:**
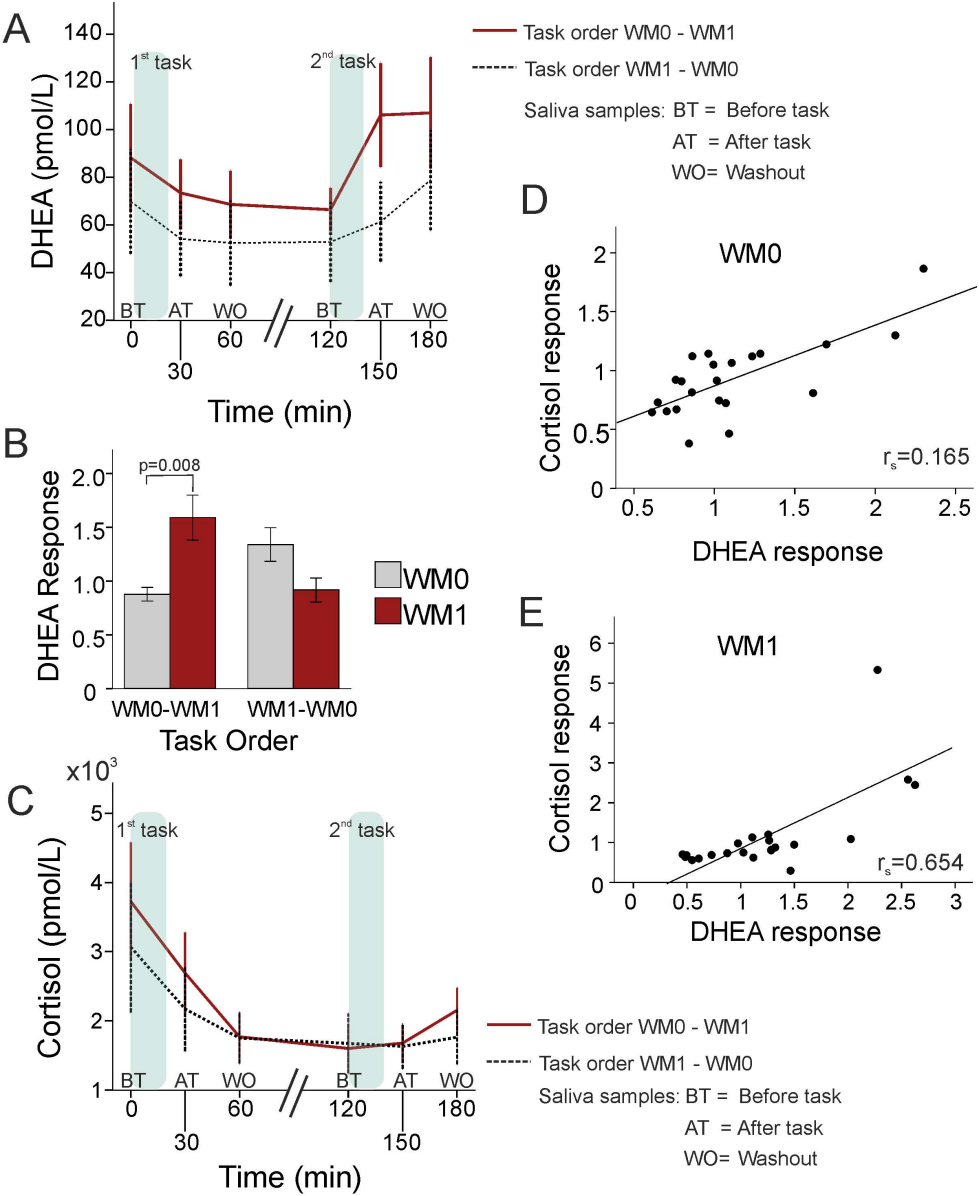
Endocrine results. A) Mean DHEA levels for each task and order. B) DHEA response for each task and order. C) Mean cortisol levels for each task and order. D) DHEA and cortisol responses (after task/before task ratios) were directly related in the discrimination task. E) DHEA and cortisol responses were directly related in the working memory task. WM0 – discrimination task; WM1 – working memory task; DHEA response: DHEA after task/before task ratio; Cortisol response: Cortisol after task/before task ratio; order WM0-WM1 – the discrimination task performed firstly and the working memory task performed secondly; order WM1-WM0 – the working memory task performed firstly and the discrimination task performed secondly. Error bars represent +/− standard error of the mean (SEM).

The waveforms elicited by standard trials in the two tasks are presented in Figure 4. The N2b significantly increased in WM1 as compared to WM0 [F_(1,21)_ = 6.738, p = 0.017; −1.8±0.9 µV in WM0 and −3.3±0.6 µV in WM1] (see figure 4A and 5B).

The analysis of visP300 revealed a task×order interaction [F_(1,21)_ = 10.184, p = 0.004], see figure 4C. Further analyses revealed a visP300 enhancement in the second task [t(22) = −3.163, p = 0.005; 2.6±0.6 µV in the first task, and 4.8±0.8 µV in the second task], independently of the WM load content of that task (figure 4D).

Overall, N2b was enhanced by WM load while the visP300 was enhanced in the second task, independently from working memory load.

### Endocrine Baseline Levels and Response

Baseline endocrine levels were: DHEA 79.0±15.5 pmol/L, DHEAS 984±120 pmol/h and cortisol 3412±622 pmol/L, with a normal distribution and no significant relation between them. These parameters were not significantly related to age and body mass index (BMI, kg/m^2^), and did not differ significantly according to the menstrual cycle phase (follicular, peri-ovulatory and luteal, based on self reported menstrual cycle day) or between subjects taking and not taking hormonal contraception. DHEA and cortisol levels during the experimental procedure are shown in Figure 5.

In contrast to DHEAS levels, which were not affected by any of the factors (task, time or task order), and therefore won’t be reported any further, repeated measures ANOVA on DHEA levels revealed a task×order interaction [F_(1,20)_ = 6.215, p = 0.022] and a task×time×order interaction [F_(2,40)_ = 9.839, p = 0.002]. Further analyses revealed that DHEA levels rose after the performance of the second task as indicated by a time effect [F_(2,40)_ = 8.415, p = 0.003; 59.6±9.4 pmol/L before task; 83.9±13.5 pmol/L after task; 92.9±15.6 pmol/L at washout], see figure 5A.

Regarding WM effects, *post hoc* analyses revealed that when the order was WM0-WM1, DHEA levels were higher after WM1 than after WM0 [F_(1,10)_ = 9.041, p = 0.013, 71.1±13.9 pmol/L after WM0 and 106.4±22.2 pmol/L after WM1]. The DHEA response [F_(1,10)_ = 10.676, p = 0.008, 0.88 for WM0 and 1.60 for WM1] was also higher in WM1 than in WM0 (figure 5B). Nevertheless, when the order was WM1−WM0, DHEA levels after the second task [F_(1,10)_ = 6.006, p = 0.034] and DHEA response [F_(1,10)_ = 4.855, p = 0.052] were similar in the WM0 (the second task) and in the WM1 task.

An repeated measures ANOVA on cortisol levels revealed a task×time×task order interaction [F_(2,40)_ = 11.809, p = 0.002]. *Post-hoc* analyses for each task order separately showed that cortisol levels decreased after WM0 when the order was WM0−WM1. In fact, for this order, there was a time effect for WM0 [F_(2,22)_ = 9.544, p = 0.007; cortisol levels were 3725±855 pmol/L before task; 2704±579 pmol/L after task; 1766±359 pmol/L at washout], see figure 5C. In spite of this, the cortisol after task/before task ratio in WM0 was not significantly different from the after task/before task ratio in WM1.

DHEA and cortisol changes were directly related for both the WM0 [after task/before task: r_s_ = 0.615, n = 22, p = 0.002, figure 5D; washout/before task: r_s_ = 0.852, n = 22, p<0.001] and WM1 task [after task/before task: r_s_ = 0.654, n = 22, p = 0.001, figure 5E; washout/before task: r_s_ = 0.656, n = 22, p = 0.001].

### Endocrine Relations to Performance

ANOVAs on hit rate and response time were used to select the endocrine parameters that covariated with performance (results are presented as supporting information, Table S1): baseline cortisol, baseline cortisol/DHEA ratio and Δ DHEA response covariated with hit rates and Δ DHEA response covariated with response times. Nevertheless, post hoc Spearman’s Rank Order correlations showed no significant relations between those endocrine parameters and performance.

### Endocrine Relations to Event-Related Potentials

#### Distraction Effects

**Figure 6:**
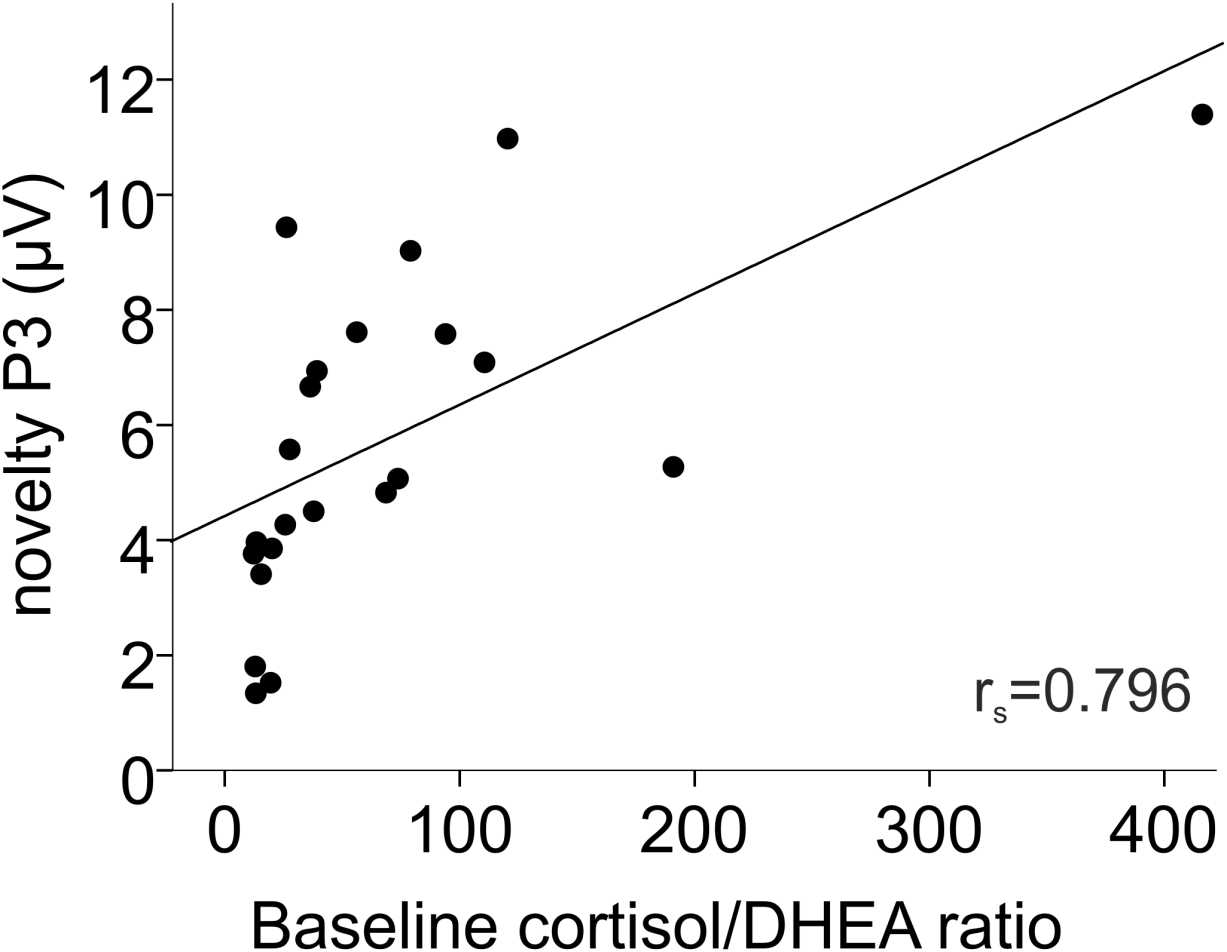
Baseline cortisol/DHEA ratio was directly related to novelty-P3 under Working Memory load.

Endocrine relations to distraction were explored by using the novel minus standard difference waves for WM0 and WM1. There were no endocrine relations to novelty-P3 in WM0. However, in WM1, the novelty-P3 amplitude was enhanced in relation to higher baseline cortisol/DHEA ratios (r_s_ = 0.796, n = 22, p<0.001; see figure 6).

#### Working Memory Effects

**Figure 7:**
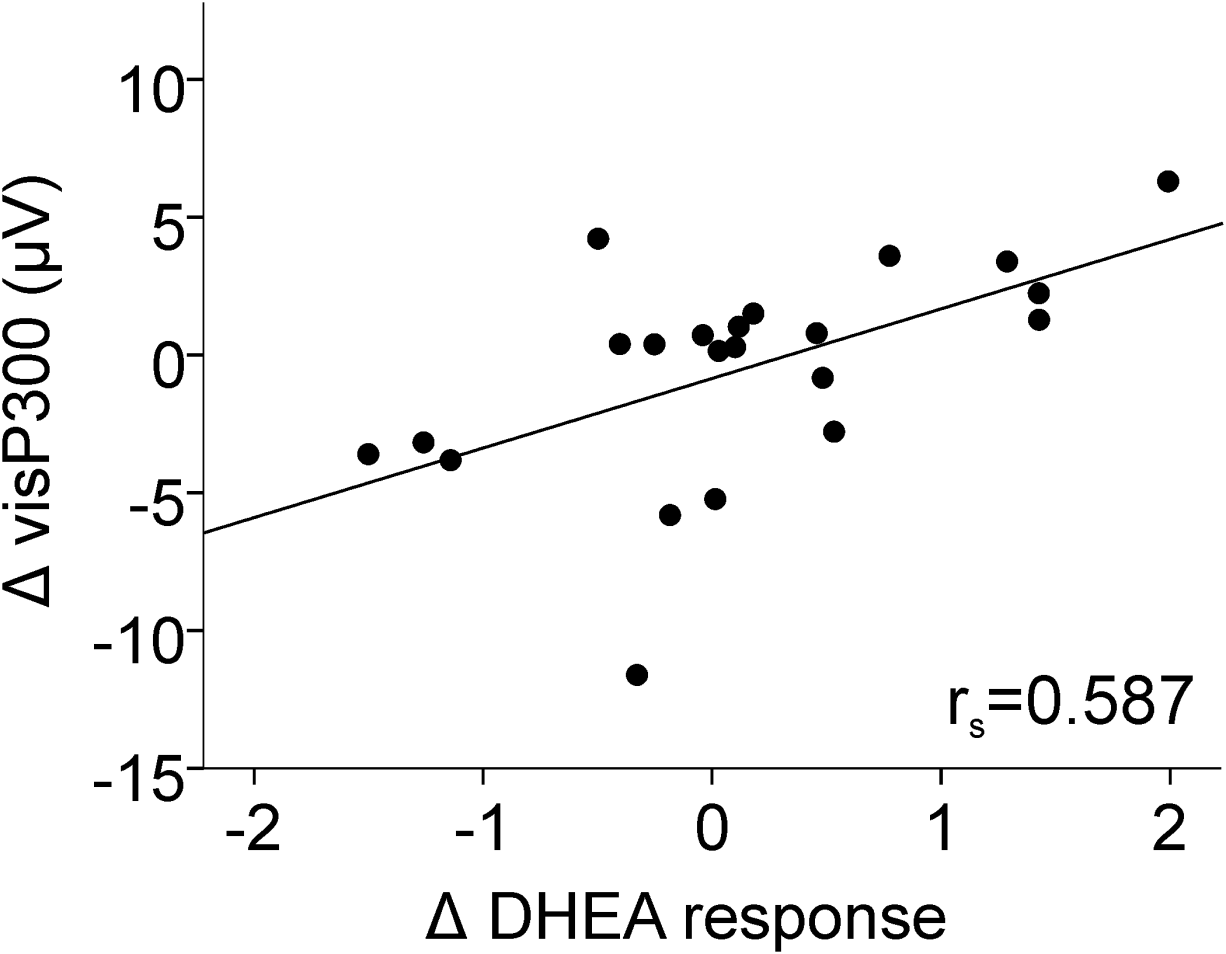
The visP300 amplitude changed between tasks (WM0, WM1) in direct relation to DHEA response. WM0 – discrimination task; WM1 – working memory task. Δ DHEA response = DHEA response in WM1 – DHEA response in WM0; Δ visP300 = mean visual P300 in WM1 - mean visual P300 in WM0.

The results showed that visP300 change between tasks was directly related to Δ DHEA response as supported by a significant interaction between visP300 amplitude×Δ DHEA response [F_(1,20)_ = 9.244, p = 0.006] and a direct relation between visP300 enhancement and DHEA response increase attributed to WM load (r_s_ = 0.587, n = 22, p = 0.004; see Figure 7). The difference of visP300 latency between tasks was not related to the endocrine parameters.

## Discussion

The present study explores the relationships between cognitive performance and endogenous DHEA, DHEAS and cortisol. As expected, the audio-visual distraction paradigm including a manipulation of working memory, yielded typical effects observed in previous studies. Indeed, the WM load task was harder to perform, as revealed by lower hit rates and longer reaction times. Likewise, novel sounds distracted the participants, as reflected by lower hit rates and longer reaction times. Regarding brain responses to novel sounds in both WM0 and WM1 tasks, the results revealed an enhanced P3 deflection, indicating that novel sounds caused distraction when compared to standards. The N2b elicited by the task-relevant visual stimuli was enhanced under WM load as expected [51].

DHEA and cortisol responses were directly related (independently of the WM load content of the task). This is in agreement with the fact that corticotrophin releasing hormone (CRH) stimulates corticotrophin secretion and in turn corticotrophin is a stimulus for DHEA secretion [59], [60], even if indirectly through the action of an unidentified DHEA androgen stimulating hormone [61], [62], [63].

Nevertheless, we found important differences between DHEA and cortisol responses with WM load manipulation. DHEA levels increased with the performance of the second task independently of the task, suggesting either a cumulative effect or a latent interval before the response. Still, the increase in DHEA levels was more pronounced with WM load. Thus, the effect of a greater cognitive effort or specific effects of WM load on DHEA levels are suggested. Interestingly, this response is specific for DHEA and does not occur with cortisol and DHEAS. In fact, regarding cortisol, a decrease was found when the subjects were performing the discrimination task (WM0). Whatever the specific mechanisms may be, there is an interesting point: the distinctive pattern of cortisol and DHEA responses. Thus, cortisol decreases after a simple task if this task comes first and DHEA increases after a second cognitive task when this is a challenging task.

Stangl et al. [64] demonstrated that DHEA administration increased DHEA levels and enhanced performance in a visual same-different task (without WM load) while cortisol levels remained constant. Also, those authors described baseline cortisol relations to performance. However, other studies failed to provide systematic evidence that DHEA and DHEA administration enhanced short-term memory at the performance level [16], [19]. In the present study, endogenous levels of DHEA, DHEAS and cortisol were measured and no significant relations were found with the accuracy or latency of the response. Nevertheless, a bigger sample of subjects may be necessary to uncover eventual relations.

On the other hand, the present study demonstrated endocrine relations to the electrophysiological recordings. In the WM task, higher baseline cortisol/DHEA ratio was related to more processing of the distracting stimuli, as indexed by an enhanced novelty-P3. This suggests that baseline cortisol enhances, whereas baseline DHEA prevents auditory distraction, and simultaneously suggests an anti-cortisol effect of DHEA. The fact that this relation became evident in the most stressful situation (WM load) agrees with previous evidence showing that DHEA has anti-cortisol effects under stress [12], [14] or that these effects are more evident under stress.

Regarding ERPs to the visual target stimuli, DHEA effects on WM load pointed towards increased visP300 amplitudes. In fact, the DHEA raise due to WM load was related with enhanced P300 amplitudes indicating enhanced memory update and suggesting a rapid DHEA behavioral effect.

Endocrine responses to stimuli are commonly used and they usually provide higher sensitivity than baseline levels to detect pathological conditions or inter-subjects differences [29], [65], [66]. As an example, glucocorticoids responses to different stimuli can be used to measure the adrenals functional reserve (predicting their response to stress) or to characterize different phenotypes of the stress response (which were related to personality traits and pathological conditions) [67], [68], [69], [70], [71]. Accordingly, in the present study, the parameter related to working memory processing was DHEA response and not baseline DHEA. Also, apart from its slow genomic effects, corticosteroids are known to have rapid non-genomic central nervous system effects [35], [39], [40].

Alternatively, the relation between visual P300 amplitude and DHEA response could suggest that subjects with enhanced working memory update are the ones with higher DHEA responses. In that regard, other authors demonstrated that chronic stress and higher cortisol levels were related with poorer memory [34], [37], [38] and reduced DHEAS response to a superimposed psychological stress [29]. Nevertheless, we found no relation between baseline cortisol and visual P300 amplitude, and therefore, an effect of chronic stress is not suggested.

Wolf et al. [43] studied the effects of DHEA replacement on short term memory ERPs. They reported an increase in P3 amplitude after DHEA replacement, which reflects an enhancement of information updating. This is in accordance with our results, as we also observed that the physiological DHEA increase was related to an enhanced visP300.

A short term increase in cortisol can damage hippocampal neurons and may impair memory [42]. This may be an oversimplification since specific types of hippocampal mediated memory may be impaired by stress but others may not [72], [73]. Yet, and hypothesizing that DHEA might prevent memory impairment under stress, Wolf et al [74] found that DHEA replacement enhanced attention but did not prevent the decline in visual memory under an acute psychosocial stress. This result did not support the idea of a direct anti-glucocorticoid effect of DHEA in hippocampal mediated memory functions. In another study DHEA protected hippocampal neurons against excitatory amino acid-induced neurotoxicity [75]. Our results also support the idea of DHEA anti-cortisol effects in distraction, but regarding working memory, we found relations with DHEA, not cortisol. Moreover, normal ranges of baseline cortisol were observed and cortisol levels did not rise with WM load, they just did not decrease.

Recent results also suggest that repeated stress and consequent activation of the glucocorticoid receptors dampens prefrontal cortex glutamatergic transmission. Actually, it facilitates glutamate receptor turnover, which has a detrimental effect on prefrontal cortex-dependent cognitive processes [76] like WM. The present results agree with the known action of DHEA on glutamatergic receptors as well as with the idea that DHEA opposes cortisol detrimental effects during the performance of working memory tasks under stress. This last relation was evidenced by inverse relations of DHEA and cortisol to distraction.

Nevertheless, working memory effects were related to the DHEA response but not to cortisol. Thus, regarding WM effects, an anti-cortisol effect of DHEA is not so evident and other specific effects of DHEA may be present. As mentioned, besides their anti-cortisol effects, DHEA has Gamma Aminobutyric Acid Type A (GABA_A_) receptor antagonism and sigma 1 agonist effects [3], [9], [10] which might underlie or contribute to the effects found. In fact, both gabaminergic antagonism and glutamatergic agonism are known to improve cognitive function.

Eventually in relation to DHEAS’ long half-life [2], [3], its levels did not change with WM load manipulation. Also, we found no relations between baseline DHEAS and performance or ERPs. Nevertheless, we found no relations between baseline DHEA and performance or ERPs either. Instead, baseline cortisol/DHEA ratio and DHEA response relations to ERPs were found.

DHEA metabolites also include other neuroactive steroids such as estradiol, estrone and testosterone [19], [77], which may mediate part of the DHEA effects, namely after DHEA administration [78]. Estrogens, in particular estradiol, enhance working memory in women [79], [80] and testosterone supplementation may enhance working memory in older men [81], [82]. We did not measure DHEA metabolites, which may mediate some of its effects. Subsequently, we cannot exclude that those steroids may contribute or eventually be responsible for the performance and electrophysiological relations we found. Finally, as our study includes only female participants, the outreach of the results is limited only to women. DHEA and DHEAS are androgens and androgen levels, namely testosterone and DHEAS levels, are higher in men than in women [2], [7]. Therefore, another group of participants would be necessary to study the electrophysiological correlates of DHEA and DHEAS in men. For further studies it would be relevant to study whether the results are identical or distinct according to gender.

In summary, a higher cortisol/DHEA ratio was related to enhanced processing of the auditory distractor during the performance of a visual working memory task. This suggests that in women, DHEA may oppose cortisol effects in involuntary distraction, reducing the processing of the auditory distractor (novelty-P3). Regarding working memory, DHEA increased with the performance of consecutive cognitive tasks, and a higher DHEA response due to WM load was related to an enhancement of the task-relevant information processing (visual P300). Overall, the results suggest that DHEA may oppose cortisol effects reducing distraction and a higher DHEA response may enhance working memory at the electrophysiological level.

## Supporting Information

Table S1 **Endocrine relations to performance.** ANOVAs of the performance parameters that covariated with endocrine measurements. Results represent the interaction between endocrine parameters and working memory (WM) condition and auditory stimulus. Baseline endocrine interactions are significant when p<0.013. Δ DHEA response = DHEA response in WM1 – DHEA response in WM0. WM0 – discrimination task; WM1 – working memory task.
(DOCX)
