# Supplementary material for: The Relationship between Dehydroepiandrosterone (DHEA), Working Memory and Distraction – A Behavioral and Electrophysiological Approach
Source: PLoS One. 2014 Aug 8;9(8):e104869. doi: 10.1371/journal.pone.0104869 (PMC4126777; doi:10.1371/journal.pone.0104869)
Supplement: Table S1 — Endocrine relations to performance. ANOVAs of the performance parameters that covariated with endocrine measurements. Results represent the interaction between endocrine parameters and working memory (WM) condition and auditory stimulus. Baseline endocrine interactions are significant when p<0.013. Δ DHEA response = DHEA response in WM1 – DHEA response in WM0. WM0 – discrimination task; WM1 – working memory task. (DOCX) [file pone.0104869.s001.docx]

|  |  | **ANOVA** |  |
| --- | --- | --- | --- |
| **Hit Rates** | | | |
| Baseline Cortisol x | Task (WM1, WM0) | F (1,21)=5.956 | p=0.024 |
|  | Sound (standard, novel) | **F(1,21)=8.222** | **p=0.009** |
|  | Task x Sound | **F(1, 21)=11.751** | **p=0.003** |
| Baseline Cortisol/DHEA ratio x | Task | **F(1,20)=8.186** | **p=0.01** |
|  | Sound | **F(1,20)=13.178** | **p=0.002** |
|  | Task x Sound | **F(1,20)=17.609** | **p<0.001** |
| Δ DHEA response x | Task | **F(1,20)=8.087** | **p=0.01** |
|  | Sound | F(1,20)=1.827 | p=0.192 |
|  | Task x Sound | F(1,20)=5.671 | p=0.027 |
| **Response times** | | | |
| Baseline Cortisol x | Task | F(1,21)=1.129 | p=0.300 |
|  | Sound | F(1,21)=4.441 | p=0.047 |
|  | Task x Sound | F(1,21)=3.038 | p=0.096 |
| Baseline Cortisol/DHEA ratio x | Task | F(1,20)=0.467 | P=0.502 |
|  | Sound | F(1,20)=6.489 | p=0.019 |
|  | Task x Sound | F(1,20)=2.104 | P=0.162 |
| Δ DHEA response x | Task | F(1,20)=3.304 | p=0.084 |
|  | Sound | F(1,20)=2.349 | p=0.141 |
|  | Task x Sound | **F(1,20)=10.734** | **p=0.004** |
